# The Necessity of Uniform Legislation

**Published:** 1883-11

**Authors:** A. O. Rawls, F. H. Rehwenkel, A. W. Aarlan

**Affiliations:** Kentucky, Committee.; Ohio, Committee.; Illinois, Committee.


ARTICLE V.

### THE NECESSITY OF UNIFORM LEGISLATION.

LEXINGTON, KY., *December* 6, 1882.

*To the President and Members of the State Board of Dental Examiners of New York :*

GENTLEMEN :—At the late meeting of the American Dental Association, held at Cincinnati in August last, there were present at least twelve members of State Examining Boards, representing five different States. An individual interchange of experience and opinion relative to methods of examinations, possible uniform standard of qualifications and other matters of import, revealed the fact that under the present state of things, it could scarcely be hoped to apply the law with anything like uniformity in the different States, on account of imperfect knowledge of many important points connected with the same, and its enforcement. In order to bring the matter into more definite shape, a regularly organized meeting was held, at which every member of an Examining Board in the city was present. Here the objects of the meeting were more fully discussed, and in furtherance of the views of those present, the undersigned were appointed a special committee. The duty of this committee is to communicate and consult with the different State Boards of Dental Examiners, to discuss all matters of importance relative thereto, and arrange for a meeting at a central point for representatives from every State which has an Examining Board.

The necessity for such meeting is certainly not within the pale of doubt, since the objects to be attained are of both special and general interest, and shared in common by our colleges, the profession, and the community at large. To state these objects briefly, it is desirous that we secure unity of effort and systematic action relative to all questions touching the manner and standard of examinations, the uniformity of the laws' action in the several States in which Dental Laws exist, the relative duties of our profession, Boards of Examiners, and Dental Colleges, and the permanent organization of a society to consist only of members of State Boards of Exaniners. Among the many questions which have already arisen, and which will, in the near future, come up more frequently and persistently, is that of receiving the certificates of qualification of other State Boads in lieu of a re-examination. Some State Laws are silent on the subject, while the laws of Illinois positively prohihit it. In behalf of those to whom is entrusted the execution of the law, in behalf of the dignity of the law, and for the sake of those amenable to the same, and seeking its protection, this latter question should be hastily and definitely settled. The more fully these matters were discussed, the more it became evident that a full meeting of representatives of the different State Boards ought to be had, so that all matters of importance might be discussed and proper conclusions arrived at.

In pursuance of the wishes of this Cincinnati meeting, the Committee would earnestly request your Board to send its duly authorized representative to a meeting to be held in the city of Lexington on the 20th day of February, 1883, beginning at 2:30 o'clock, and continuing one or more days, as necessity may demand. Headquarters, Phœnix Hotel.

A. O. RAWLS, Kentucky,
F. H. REHWENKEL, Ohio,
A. W. AARLAN, Illinois,
Committee.

